# Yielding behaviour of chemically treated *Pseudomonas fluorescens* biofilms

**DOI:** 10.1016/j.bioflm.2024.100209

**Published:** 2024-07-03

**Authors:** Samuel G.V. Charlton, Saikat Jana, Jinju Chen

**Affiliations:** a Department of Civil, Environmental and Geomatic Engineering, Institute of Environmental Engineering, ETH Zürich, Zürich, 8093, Switzerland; b Ulster University, School of Engineering, 2-24 York Street, Belfast, BT15 1AP, United Kingdom; c Newcastle University, School of Engineering, Newcastle Upon Tyne, NE1 7RU, United Kingdom; d Loughborough University, Department of Materials, Loughborough, LE11 3TU, United Kingdom

## Abstract

The mechanics of biofilms are intrinsically shaped by their physicochemical environment. By understanding the influence of the extracellular matrix composition, pH and elevated levels of cationic species on the biofilm rheology, novel living materials with tuned properties can be formulated. In this study, we examine the role of a chaotropic agent (urea), two divalent cations and distilled deionized water on the nonlinear viscoelasticity of a model biofilm *Pseudomonas fluorescens*. The structural breakdown of each biofilm is quantified using tools of non-linear rheology. Our findings reveal that urea induced a softening response, and displayed strain overshoots comparable to distilled deionized water, without altering the microstructural packing fraction and macroscale morphology. The absorption of divalent ferrous and calcium cations into the biofilm matrix resulted in stiffening and a reduction in normalized elastic energy dissipation, accompanied by macroscale morphological wrinkling and moderate increases in the packing fraction. Notably, ferrous ions induced a predominance of rate dependent yielding, whereas the calcium ions resulted in equal contribution from both rate and strain dependent yielding and structural breakdown of the biofilms. Together, these results indicate that strain rate increasingly becomes an important factor controlling biofilm fluidity with cation-induced biofilm stiffening. The finding can help inform effective biofilm removal protocols and in development of bio-inks for additive manufacturing of biofilm derived materials.

## 1 Introduction

Bacterial biofilms are communities of bacteria organised within a self-secreted extracellular polymeric matrix (ECM) [1]. Biofilms are ubiquitous in the environment and develop under a wide range of physicochemical conditions and mechanical forces [2,3]. The ECM serves as a structural scaffold, facilitating cohesive interactions among the constituent cells and ECM polymers [4]. Typically, the ECM is composed of polysaccharides, proteins, and eDNA, although the relative abundance of each component can vary considerably as a function of bacterial species [5,6]. The interaction between the ECM components play a crucial role in shaping a biofilm's mechanical properties, architecture, and biological function [[6], [7], [8], [9], [10]]. Despite the diversity of extracellular polymers and cell shapes within biofilms, they can be conceptually considered similar to colloidal polymer systems [11,12]. Akin to colloidal polymer materials biofilms are viscoelastic [13,14]. Viscoelasticity is a key factor in biofilm proliferation, allowing for the dissipation of internally generated and externally applied stresses through the rearrangement of the ECM-cell network [13,15,16]. The balance between the elastic and viscous components determines biofilm morphogenesis in the presence of shear stress [17]. For example, the elastic contribution impacts the recoverability and permeability of biofilms in membrane filtration systems, while biofilm viscosity influences the development of biofilm streamers [18,19]. The viscoelasticity of biofilms can also be altered due to exposure to ionic fluctuations or changes in pH, which mediate interaction forces within the cell-ECM network [20]. Depending on nature and valency of the ionic charges biofilm can stiffen, weaken or show enhanced stability against erosion in shear flows [[21], [22], [23]]. The ability to modulate the viscoelasticity of biofilms has been utilized to investigate effective biofilm removal strategies [24,25] and develop biofilms as functional glues or 3D printed materials [26,27].

Biofilms in natural environments, industrial bioreactors and 3D bio-printing are subjected to large shear strain and rates [[28], [29], [30]]. In the large strain regime, a structural evolution occurs within the biofilm network resulting in non-linear viscoelastic behaviour. The non-linearity in material behaviour reflects the biofilm's ability to store or dissipate energy as its microstructure is broken down. Quantifying this dynamic is important for understanding fouling and clogging in natural and engineered systems [31]. In a range of soft materials, non-linear viscoelastic measurements have provided insights into how underlying structure, interactions, etc. influence the viscoelastic behaviour upon the application of large strains [[32], [33], [34], [35]]. Within these experiments the material microstructure is modulated by varying physicochemical parameters such as ionic concentration, packing fraction etc. and the emergent nonlinear viscoelastic response is investigated by using various phenomenological models [[36], [37], [38], [39]].

In this paper, we investigate the viscoelastic behaviour of *Pseudomonas fluorescens* biofilm subjected to two divalent cations, Calcium (Ca^2+^), Ferrous (Fe^2+^) and a chaotropic agent, urea (NH_2_CONH_2_). Using nonlinear rheological analysis tools, the effects of chemical treatments on the biofilm viscoelasticity is investigated. We reveal how a biofilms rheology is altered by chemical modification resulting in a yielding transition from strain softening to strain rate thinning upon exposure to divalent cations. The emergence of three characteristic yielding behaviours and their rheological origins are investigated using confocal microscopy and rheometry. By applying high-fidelity Large Amplitude Oscillatory Shear (LAOS) techniques, we can capture subtle changes to the yielding behaviour in biofilm. The presented results enable a comprehensive understanding of chemical treatments on biofilm rheology and can be used to inform the development of new removal strategies or for the development of bio-inks for 3D printing of biofilms.

## 2 Methods and materials

### 2.1 Bacteria culture and biofilm growth

For rheology and imaging experiments *P. fluorescens* (DSM 50090) was grown overnight from glycerol stocks in LB broth (Lennox, Sigma) at 24°*C* with shaking (150 rpm). Overnight cultures of *P. fluorescens* 250 μL) were spread onto 1.5 % Nutrient Broth (NB) (Sigma, UK) agar plates (diameter = 90 mm, agar volume = 20–25 ml) with an L-shaped spreader. The NB agar plates were then incubated at 24°*C* for 72 h. The chemical treatment was applied by spreading a 1 ml liquid layer of 100 *m*M FeCl_2_, 100 *m*M CaCl_2_, 100 *m*M urea or distilled deionized water (ddH_2_O) across the biofilm developed on the agar plate. After 60 min, the excess of the treatment solution was removed by tilting the plate. The biofilm was then incubated for 30 min at 24°*C* before collection. The treated biofilm samples were transferred to the bottom plate of a rheometer by gentle scraping with a microscope slide.

### 2.2 Linear rheology

A Malvern Kinexus Pro + rheometer (Malvern, UK) mounted on a passive isolation plate (Terra line #1580-23) was used for rheological measurements. The rheometer was operated in a strain-controlled configuration. A 20 mm parallel plate geometry was used and adhesive grit paper (120 grade) attached to double sided tape was applied to the top and bottom surface of plates to minimize the slip. A built-in peltier element was used to maintain the temperature of 24°*C* at the bottom plate. The biofilm samples were placed on the bottom plate with the gap height of 1 mm and a normal force of approximately 0.1 N was set at the beginning of the measurement. A biofilm volume of approximately 300 μL was used for each measurement and excess sample was trimmed using a razor blade. A solvent trap (KNX2513, Malvern, UK) was used to minimize the effect of desiccation during measurements. Frequency sweeps were performed on the samples from 0.1 to 50 Hz at a strain amplitude of 1 %. The viscous (*G*″) and elastic modulus (*G*′) and phase angle (*δ*) were measured across this frequency range.

### 2.3 Non-linear rheology

Nonlinear rheological analysis was performed using Fourier Transform coupled Chebyshev polynomial analysis implemented in MITlaos [40] and the Sequence of Physical Processes (SPP) [41]. While both techniques quantify the nonlinear viscoelastic response, MITlaos calculates rheological descriptors at specific points (i.e. minimum and maximum applied strain) on a Lissajous curve while SPP offers a continuous description of material states along a Lissajous curve. Rheometer raw data were acquired for 24 strain (*γ*) amplitudes logarithmically spaced between 0.1 % and 250 % at a frequency (*ω*) of 1 Hz. The raw data comprised time, angular displacement, and torque and was sampled at 5 kHz. For each strain amplitude, 17 oscillatory cycles were performed. The raw data were pre-processed using a custom Matlab routine. Boxcar filtering was used to smooth the raw signal, with a window width of 100 points. Finally, a subset of 5 cycles identified as in steady state were extracted from each strain amplitude dataset. The Chebyshev analysis was performed using MITlaos and intracycle thickening/thinning (*T*) and intracycle stiffening/softening (*S*) ratios were calculated. The thickening ratio (*T* > 0 or *T* < 0) quantified viscous thickening/thinning and is the ratio of the maximum strain-rate dynamic viscosity $\eta_{L}^{\prime }$ to the minimum strain-rate dynamic viscosity $\eta_{M}^{\prime }$. The large rate viscosity $\left(\eta_{L}^{\prime }\right)$ is calculated from Lissajous- Bowditch plots as $\eta_{L}^{\prime }$ = ${\left|\frac{d\sigma }{d\dot{\gamma }}\right|}_{\dot{\gamma }={\dot{\gamma }}_{\max}}$. The minimum rate dynamic viscosity, $\eta_{M}^{\prime }$ is calculated as $\eta_{M}^{\prime }$ = ${\left|\frac{d\sigma }{d\dot{\gamma }}\right|}_{\dot{\gamma }=0}$ (Supplementary Fig. S1). The stiffening ratio (*S* > 0 or *S* < 0) quantified the intracycle elastic stiffening/softening, which is the ratio of the maximum strain elastic modulus $G_{L}^{\prime }$ to the minimum strain elastic modulus $G_{M}^{\prime }$. Here the large strain elastic modulus, $G_{L}^{\prime }$ is calculated from Lisaajous-Bowditch plots as $G_{L}^{\prime }$ = ${\left|\frac{d\sigma }{d\gamma }\right|}_{\gamma =\gamma_{\max}}$ and the minimum strain elastic modulus, $G_{M}^{\prime }$ is calculated as $G_{M}^{\prime }$ = ${\left|\frac{d\sigma }{d\gamma }\right|}_{\gamma =0}$ (Supplementary Fig. S1). The non-linear parameter *I*_3/1_ was calculated as a ratio of the intensity of the third harmonic to the fundamental harmonic. The emergence of a characteristic slope in log-log plot of *I*_3/1_ vs. *γ* was utilized to quantify the transition from linear to nonlinear behaviour in a LAOS sweep. $G_{\mathrm{norm}}^{\prime \prime }$(*γ*) was calculated by normalising *G*′′(*γ*) by the *G*″ value obtained from the linear viscoelastic region of each of the respective biofilm.

The sequence of physical processes (SPP) analysis was performed using the SPPplusv1 MATLAB routine, kindly provided by Prof. Simon Rogers. SPP analysis uses the Frenet-Serret (TNB) reference coordinate frame to map [*γ*, $\dot{\gamma }$, *σ*] into the transient elastic $\left(G_{t}^{\prime }\right)$ and transient viscous modulus $\left(G_{t}^{\prime \prime }\right)$ [41]. The MATLAB routine outputs the instantaneous transient elastic and viscous modulus from thich the transient phase angle was calculated as $\delta_{t}=\tan^{-1}\left(\frac{G_{t}^{\prime \prime }}{G_{t}^{\prime }}\right)$. During each strain cycle the biofilms initially behaved elastically, with stress being linear with strain. To describe this part of the cycle we use the residual modulus, *G*_*R*_, which is calculated using, $G_{R}={\left|\frac{d\sigma }{d\gamma }\right|}_{\sigma =0}$ (Supplementary Fig. S2). The accumulated strain, *γ*_*accumulated*_ denoted the total strain accumulated at the point of maximum stress within the biofilm from the lower reversal point (where *γ* is at a minimum). For each chemical treatment at least three biological replicate measurements were performed and the above measures were calculated.

### 2.4 Bright-field and confocal microscopy

Bright-field microscopy was performed on biofilms after the respective chemical treatment. The agar plates were inverted and placed on the stage of an inverted bright-field microscope (Nikon Ti–S). Images were acquired with a 10x objective using a camera (GS3-U3-51S5M, Grasshopper). For confocal imaging, after treatments with the chemical compounds, the biofilms were stained in-situ using 10 μL of 10 μM Syto63 (Sigma, S11345) and incubated for 30 min. After staining, agar supporting the biofilm samples which had been stained were cut using a scalpel blade and carefully transferred to a microscope slide attached to a 25 μL geneframe (Sigma, UK). The geneframe was sealed with a #1.5 coverslip. Imaging was performed on a Leica SP8 confocal laser scanning microscope (CLSM) using a 100x oil immersion objective. Syto63 was excited at a wavelength of 660 nm and the emission filter was set between 670 and 750 nm. A total of 5 random fields of view (FOV) measuring 101 μm × 101 μm were imaged at 1024 pixels × 1024 pixels, yielding a pixel calibration of 98.6 nm/pixel. The pinhole was set at 1 Airy units (AU). Imaging was performed 10 μm above the coverslip. Images were pre-processed in ImageJ by applying a contrast-limited adaptive histogram equalization (CLAHE) filter with settings: box size = 127 pixels, block size = 50, histogram = 256, maximum = 2, for denoising and contrast enhancement [9]. Image smoothing was performed with a Gaussian filter of sigma of 2 pixels and then a Laplacian of Gaussian filter with a radius of 2 pixels before binarization using the Otsu thresholding method. The packing or area fraction (*φ*) was then calculated as the ratio of cells to void space using relation *φ* = *A*_*cells*_/*A*_*void*_.

### 2.5 Statistical analysis

Data are represented as mean values with standard errors. Statistical differences between samples were determined by one-way student T-tests. P < 0.05 was considered statistically significant in this study, as indicated by the symbols in the representative figures.

## 3 Results

### 3.1 Biofilm microstructural features and rheology are altered due to chemical treatments

**Fig. 1:**
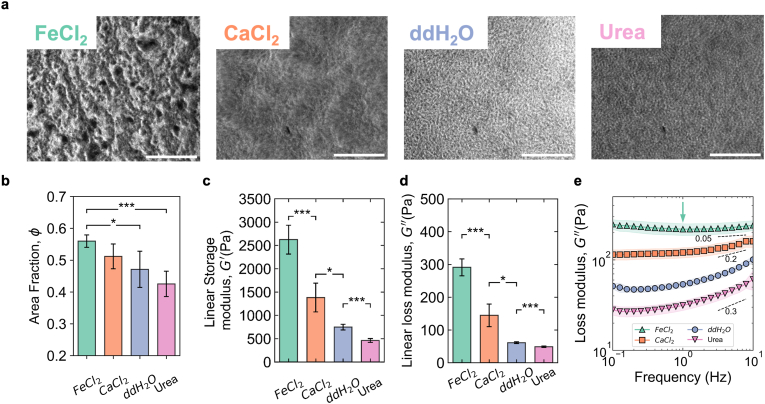
**Treatment with divalent cations and urea modifies the microstructure and viscoelastic modulus of *P. fluorescens* bacterial biofilms.** (**a**) The macroscopic morphology of biofilm lawns on agar plates alters with chemical treatment. Representative bright field images of each bacterial biofilm after chemical treatment. The scale bars are 250 μm. (**b**) The packing fraction of each biofilm due to the applied chemical treatment (*n* ≥ 3) (**c**) The elastic modulus of each biofilm taken from within the linear viscoelastic regime (*n* ≥ 3). (**d**) The viscous modulus of each biofilm taken from within the linear viscoelastic regime (*n* ≥ 3). (**e**) Frequency sweep of the chemically treated biofilms, the dashed line and gradient indicate the frequency dependence of each biofilm (*n* ≥ 3). Shaded region indicates standard deviation of the mean.

Application of divalent cation and urea treatments caused alterations to the microstructural features and packing fraction of bacterial biofilms (Fig. 1a). The microstructure was characterised from confocal images (Supplementary Fig. S3) in terms of packing fraction (*φ*, defined in section 2). Treatment with urea resulted in biofilms with the lowest *φ*, and regions of void space surrounding clusters of bacteria (0.43 ± 0.04, Fig. 1b). DdH_2_O treated biofilms had a marginally higher packing fraction (0.47 ± 0.06) and also had small void spaces. In contrast, the chemical treatments of CaCl_2_ and FeCl_2_ resulted in the highest *φ* and were absent of noticeable voids (0.51 ± 0.03 and 0.55 ± 0.02, respectively). The analysis revealed that addition of cations modified the cell-cell or cell-ECM interactions within biofilm matrix and lead to an increased packing fraction of the bacteria within biofilms. The overall packing fraction of ddH_2_O and divalent cation treated biofilms were within the range of crystal state for spherical colloids [42] and remained below the glass packing fraction for rods [43].

Treatment with chemical solutions modified the biofilm's viscoelastic properties and frequency dependence. Biofilms grown on agar had *G*′ values of 2200 ± 137 Pa (Supplementary Fig. S5). To dissect the role of chemicals, we considered ddH_2_O treated biofilms as the control, so that the hydration and solids content of all tested biofilms remain similar. The elastic moduli of our reference biofilm (ddH_2_O treated) had a higher linear elastic modulus (*G*′ ∼ 0.6 kPa) when compared to biofilms grown in fully submerged condition (*G*′ ∼ 10 Pa) [44,45]. Regardless of the treatments, all of the biofilms displayed an elastically dominant rheology across the probed frequency range (elastic modulus (*G*′) > viscous modulus (*G*″), Fig. 1c and d, Supplementary Fig. S4). Addition of ddH_2_O to agar grown biofilms hydrated the ECM network, resulting in a *G*′ of approximately 590 ± 54 Pa (Fig. 1c). Treatment with divalent cations (CaCl_2_ and FeCl_2_) strengthened the elastic modulus of the biofilms (1100 ± 186 Pa and 1600 ± 129 Pa, respectively) when compared to the ddH_2_O. However, urea had a weakening effect and caused a reduction of the elastic and viscous modulus (*G*′ = 360 ± 56 Pa, *G*′′ = 60 ± 15 Pa, Fig. 1d). The viscoelastic behaviour of ddH_2_O and urea biofilms was frequency dependent (dashed lines, Fig. 1e), an emergent behaviour which can be induced in hydrogels by reducing polymer crosslinking density [46]. The coefficient *k* of the power law dependency (*Ax*^*k*^) was ∼0.3 for urea or ddH_2_O treated biofilms and ∼0.2 for CaCl_2_ treatment. Contrary, FeCl_2_ treatment to biofilms induced a *G*″ minimum, and frequency independence (k ∼ 0.05) a feature seen in attractive gel systems (Fig. 1e, green arrows) [47].

### 3.2 Divalent cation and urea treatments alter biofilm yielding modes

**Fig. 2:**
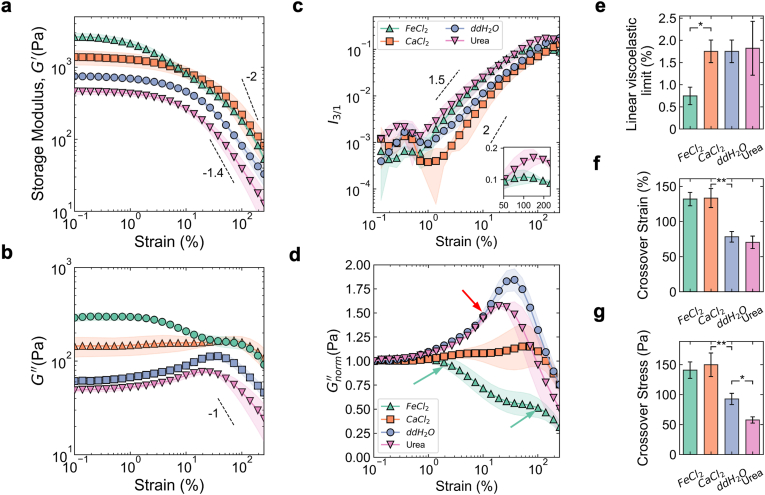
***P. fluorescens* biofilms show weak strain overshoot and/or two-step yielding behaviour depending upon the chemical treatment.** (**a**) The elastic modulus (*G*′) of each biofilm during an amplitude sweep (*n* ≥ 3). Shaded region indicates standard deviation of the mean. (**b**) The viscous modulus (*G*″) of each biofilm during an amplitude sweep (*n* ≥ 3). Shaded region indicates standard deviation of the mean. (**c**) The non-linear viscoelastic parameter *I*_3/1_ as a function of applied strain (*n* ≥ 3). Inset depicts the maximum, then drop in *I*_3/1_ for biofilms treated with FeCl_2_ and Urea. Shaded region indicates standard deviation of the mean. (**d**) $G_{\mathrm{norm}}^{\prime \prime }$ as a function of applied strain (*n* ≥ 3). Shaded region indicates standard deviation of the mean. (**e**) The linear viscoelastic region of each amplitude sweep (*n* ≥ 3). (**f**) The cross-over stress of each biofilm, calculated from the intersection of *G*′ and *G*″ in the amplitude sweep (*n* ≥ 3). (**g**) The cross-over strain of each biofilm corresponding to the calculated cross-over stress (*n* ≥ 3).

The yielding behaviour of *P. fluorescens* biofilm transitioned as a function of chemical treatment. To probe the yielding behaviour of each biofilm we performed amplitude sweeps (Fig. 2a and b). The nonlinear parameter (*I*_3/1_) was used to identify the linear viscoelastic limit. The linear viscoelastic limit is identified from the emergence of a power law exponent from the log-log plot of non-linear parameter vs. applied strain (Fig. 2c). The linear viscoelastic limit was unchanged by the treatments, however, the power law exponent for each biofilm varied as a function of chemical treatment. For the “weakest” and “strongest” biofilms (urea and FeCl_2_, respectively) the non-linear parameter increased, reached a peak and then declined (inset, Fig. 2c), indicating structural breakdown and a liquid-like behaviour [47]. In contrast, the ddH_2_O and CaCl_2_ treated biofilms displayed a constant increase in the non-linear parameter throughout the applied strain range (0 − 250 %).

Viscous modulus exhibited a chemical treatment dependent emergence of weak strain overshoot. Beyond the linear viscoelastic limit and before fluidization (denoted by the crossover strain), the biofilms displayed varying viscous *G*″ dissipation dynamics, these are represented by the $G_{\mathrm{norm}}^{\prime \prime }$ (see section 2, Fig. 2d). The biofilms treated with ddH_2_O and urea displayed weak strain overshoots (red arrows, Fig. 2d). Weak strain overshoots are hypothesized to occur due to transition from solid-like viscoelastic dissipation to fluid, plastic deformation [48]. The peaks of $G_{\mathrm{norm}}^{\prime \prime }$ indicates the resistance to flow alignment, and was lower in urea-treated than the ddH_2_O-treated biofilms (1.57 ± 0.01 vs 1.84 ± 0.12). Weak strain overshoots have been observed across different biofilm species [10,13,27]. Divalent cation treatments altered the *G*″ evolution as a function of applied strain. The FeCl_2_ biofilms exhibited a drop in *G*″ for *γ* ∼ 2 %), then plateaued (10 % ≤ *γ* ≤ 100 %) and decreased again (*γ* > 100 %), suggesting two distinct yielding regimes (green arrows, Fig. 2 d). This dissipation behaviour resembles that of colloidal gel systems with short-range interparticle attractions, where a two-step decrease in *G*″ is observed [38,49]. In contrast to the other treatments, CaCl_2_ biofilms were absent from overshoots, and didn't feature two-step yielding. The crossover stress (*σ*_*c*_) and crossover strain (*γ*_*c*_) for ddH_2_O and urea-treated biofilms was significantly lower than divalent cations treated biofilms (Fig. 2f and g). The divalent cation treated biofilms had a shear thinning behvaiour with a power law response of *G*′ ≈ *γ*^−2*n*^ and *G*′′ ≈ *γ*^−*n*^, where n is the power law gradient (Fig. 1d). The ddH_2_O and urea biofilms had an exponent ratio n′/n'′ < 2 (see lines/arrows in figure(2 a,b)).

### 3.3 Chemical treatment alters biofilm intra-cycle thickening and softening characteristics

**Fig. 3:**
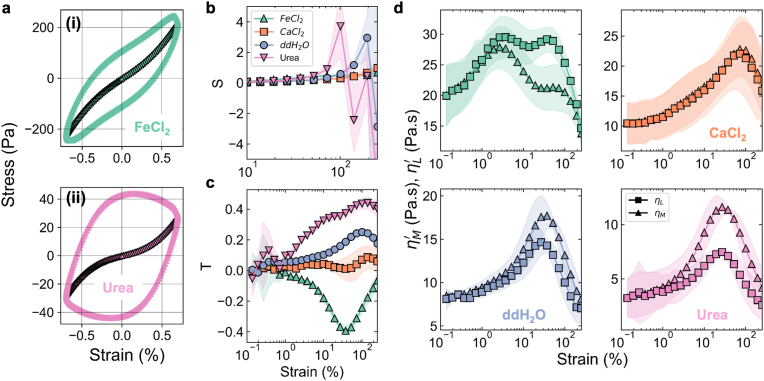
**The intracycle thickening behaviour of bacterial biofilms is altered in response to chemical treatment.** (**a**) Elastic Lissajous Bowditch plots for the (i) FeCl_2_ and (ii) Urea treatments. Shown are the representative plot at *γ* = 52 %. (**b**) The intracycle strain stiffening parameter (*S*) as a function of applied strain (*n* ≥ 3). Shaded region indicates standard deviation of the mean. (**c**) The intracycle shear thickening ratio (*T*) as a function of applied strain (*n* ≥ 3). Shaded region indicates standard deviation of the mean. (**d**) The minimum rate $\left(\eta_{M}^{\prime }\right)$ and large rate viscosity $\left(\eta_{L}^{\prime }\right)$ vs. applied strain for different chemical treatments on biofilms (*n* ≥ 3). Shaded region indicates standard deviation of the mean.

The linear to non-linear transition was altered by chemical treatments with divalent cations and urea. The transition revealed changes in the elastic (*σ* vs *γ*) and viscous (*σ* vs $\dot{\gamma }$) Lissajous - Bowditch (LB) plots which were used to map the intra-cycle dynamics (Fig. 3a(i), a(ii)). Within the linear viscoelastic region, the elastic LB plots remained elliptical and with increasing *γ* the LB plots for each biofilm began to deviate from the elliptical shape. At large strains the elastic LB plots became rectangular with increasing *γ* (Supplementary Fig. S6), a trait seen in fully yielded elastoplastic materials [52]. The ddH_2_O and urea treatments induced intersecting secondary loops in the viscous LB plots (Supplementary Fig. S7, red arrow), signifying stress overshoots where stress is dissipated quicker than accumulation of strain [53].

The intracycle shear thickening ratio (*T*) and the intracycle strain stiffening ratio (*S*) indicated rheological changes in the biofilm as a function of chemical treatment and applied strain (see methods, (Fig. 3b and c). In the linear viscoelastic regime (LVER), both T and S are ≈ 0. For *S* > 0 and *T* > 0 the biofilms exhibit strain stiffening and shear thickening, respectively, and for *S* < 0 and *T* < 0 the biofilms are strain softening and shear thinning behaviour. All biofilms experienced strain stiffening and the dynamics of strain stiffening varied with the chemical treatment (Fig. 3b). The *S* for biofilms treated with ddH_2_O and urea rapidly increased to reach maximal values of 2.9 and 3.7 and the peaks occurred at an applied strain of *γ* ∼ 139 % and *γ* ∼ 72 %, respectively (Fig. 3b). In contrast, the divalent cation-treated biofilms had a lower maximum value of *S* ∼ 0.9 and the peak occurred at strain amplitudes of *γ* ∼ 250 %.

The minimum strain viscosity, $\eta_{M}^{\prime }$ and the large strain viscosity, $\eta_{L}^{\prime }$ revealed a treatment-dependent transition in intracyle non-linear thickening or thinning. Beyond the linear viscoelastic limit, biofilms treated with ddH_2_O and urea displayed strain dependent thickening or thinning behaviour ($\eta_{L}^{\prime }$ < $\eta_{M}^{\prime }$) (Fig. 3d, lower panels). However, the divalent cation treatments altered the $\eta_{M}^{\prime }$ and $\eta_{L}^{\prime }$ characteristics. For biofilms treated with CaCl_2_, $\eta_{M}^{\prime }$ and $\eta_{L}^{\prime }$ increased or decreased at the same rate, indicating an equal contribution of strain and strain-rate dependent thickening (Fig. 3d, top-right panel). The biofilms treated with FeCl_2_ diverged from CaCl_2_ at strain greater than 1 % and displayed a predominance of rate dependent thinning ($\eta_{L}^{\prime }$ > $\eta_{M}^{\prime }$). The large strain viscosity $\eta_{L}^{\prime }$ for FeCl_2_ treated biofilms had a double peak (Fig. 3d, top-left panel). Both of the $\eta_{L}^{\prime }$ peaks coincided with drops in $\eta_{M}^{\prime }$. Together these two features suggest the presence of two yielding length-scales in the FeCl_2_ treated biofilms.

### 3.4 Visualising chemically dependent biofilm yielding modes

**Fig. 4:**
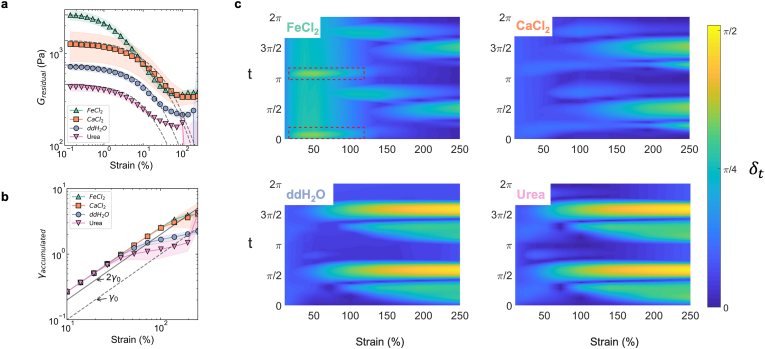
**The transient phase angle reveals the emergence of two step yielding for biofilms treated with divalent cations** (**a**) The residual modulus, *G*_*R*_ for each of the biofilms as a function of strain amplitude (*n* ≥ 3). Shaded region indicates standard deviation of the mean. (**b**) The accumulated strain for each of the biofilms. The gradient 2*γ*_0_ denotes elastically accumulated strain and divergence to a slope of *γ*_0_ indicates accumulated strain due to purely viscous effects (*γ*_0_ is the oscillatory strain amplitude, *n* ≥ 3). Shaded region indicates standard deviation of the mean. (**c**) The transient phase angle, *δ*_*t*_ as a function of strain and time within an amplitude cycle. t = *π*/2 and 3*π*/2 indicate the points of maximum strain amplitude and t = 0 and 2*π*, the point where strain rate is maximum. The red dashed box highlights the emergence of a first yielding band, which precedes a second yielding band. Shown are representative plots for each of the treated biofilm.

Biofilms are analogous to colloidal systems, the viscoelastic response of such systems are a function of inter-particle interactions and ECM mediated interactions [50,54]. To probe the stress response of such systems the concept of cage modulus (*G*_*cage*_) in colloidal glasses or residual modulus (*G*_*R*_) in colloidal gels have been previously used [55,56]. These measures broadly describe the deformation, breakage and flow of a cage like or a percolated network within a material, in response to applied strain. The initial elastic response of biofilms was quantified using $G_{R}=\frac{d\sigma }{d\gamma }{|}_{\sigma =0}$ (see methods) after a strain oscillation cycle [55]. Within the LVER, *G*_*R*_ and *G*′ were equal as no significant structural rearrangements occurred in the biofilms. Beyond the LVER, for each biofilm *G*_*R*_ reduced with increasing strain and the rate of decrease was largest for FeCl_2_ treatment (Fig. 4a). *G*_*R*_ plateaued at *γ* ≥ 75 % for ddH_2_O and divalent cation treated biofilms, reflective of the similarities in structure between biofilms. Next, we compare the accumulated strain *γ*_*accumulated*_ of each biofilm defined as the cumulative intracycle strain acquired from the minimum strain to the maximum stress values within a LB curve (Supplementary Fig. 2). Strain accumulation due to elastic effects was indicated by a gradient of 2*γ*_0_ whilst a slope of *γ*_0_ indicated strain accumulation from viscous effects. Biofilms treated with divalent cations showed an elastic dominated stress accumulation up to *γ*_0_ ≈ 100 % (Fig. 4b). Whilst, the urea and ddH_2_O-treated biofilms diverged away from elastic accumulation at *γ*_0_ > 25 %. Together these observations indicate that ddH_2_O and urea treatments uniquely modify the biofilm structure and the strain accumulation when compared to the divalent cations.

Further insights into biofilm-yielding behaviour as a function of chemical treatment was obtained using the heat maps of transient phase angle, *δ*_*t*_ (Fig. 4c) as a function of *γ* and time (t). The magnitude of the transient phase angle, *δ*_*t*_ for the ddH_2_O and urea-treated biofilms were similar with with the exception of marginally higher *δ*_*t*_ at *γ* < 25 % for the urea treatment. Both biofilms showed two major and two minor yielding bands where $\delta_{t}>\frac{\pi }{4}$, at high strain amplitudes i.e. *γ*. For both biofilms, the major yielding bands approached $\delta_{t}∼\frac{\pi }{2}$, corresponding to a fully yielded and viscous dominated response. The major yielding bands were centered around transient phase angle regions of maximum strain corresponding to *t* = *π*/2 or *t* = 3*π*/2. This indicated that the yielding of these biofilms was primarily strain-dependent. The *δ*_*t*_ behaviour of FeCl_2_ treated biofilms revealed the emergence of two yielding regions, one at low strain (red boxes, Fig. 4c), and a second at high strain. The magnitude of *δ*_*t*_ was substantially higher in the low strain yielding band for FeCl_2_ treated biofilm than the CaCl_2_ treated biofilm. This indicated that FeCl_2_ impacted cell-cell or cell-ECM interactions to a larger degree than CaCl_2_. For FeCl_2_ treated biofilms, the low strain yielding zone was strain rate dependent (peaks of *δ*_*t*_ occurred at *t* = (0, *π*)). For both FeCl_2_ and CaCl_2_ biofilms the high strain yield zone was predominantly strain dependent (at *t* = *π*/2 and *t* = 3*π*/2). Together, the transient phase angle analysis of divalent cation treated biofilms indicated a susceptibility to strain rate yielding at low strain values and a distinct partitioning of yielding into two regions at low and high strain amplitudes.

## 4 Discussion

In this study, we investigated the influence of chemical agents on the yielding behaviour of *P. fluorescens* biofilm. We applied concepts of non-linear rheological analysis to probe the alterations in large strain-yielding behaviour. This enabled us to uncover a transition from strain overshoots in the ddH_2_O biofilm to two-step yielding caused by treatment with metal ions. Our results indicated that exposure to certain metal ions, can cause biofilm strengthening which coincides with a transition to increased susceptibility to strain rate yielding.

The cell surface of the gram-negative bacterium *P. fluorescens* consists of negatively charged lipopolysaccharides, and proteins such as LapA which initiate biofilm formation on surfaces [57,58]. Akin to colloidal systems, the addition of divalent cations in our biofilm system could regulate electrostatic interactions between bacterial cells or between the cell and the ECM [49,51]. In *P. fluorescens* the ECM's exopolysaccharide component predominantly comprises of glucuronic and guluronic acids, along with rhamnose, glucose, and glucosamine [59]. Thus, the ECM has an alginate-dominated composition. Divalent cations like Ca^2+^ and Fe^2+^ are known to promote crosslinking within alginate gels through the “egg-box model”, which describes the ion-mediated chain-to-chain association between blocks of glucuronic and guluronic acid [60]. We hypothesize that the combination of these interactions within the cell-ECM network alters the viscoelastic behaviour of biofilms treated with divalent cations compared to those treated with the control and urea.

Divalent and trivalent metallic ions have been known to bind to protein and polysaccharide structures resulting in the formation of composite materials and/or self-healing bonds [61]. The major components of *P. fluorescens* ECM are alginate, uronic acid polysaccharide and the LapA adhesin protein [57]. Our findings suggest that the addition of metal ions increases the elasticity of *P. fluorescens* biofilms and alters their yielding mode in large shear regime. The stiffening effect of FeCl_2_ has been documented across several biofilms, demonstrating its ability to increase biofilm stability and erosion resistance under fluid shear [23,62]. Although exposure to metal ion FeCl_2_ increases elasticity, it concurrently renders the biofilm susceptible to strain rate yielding. Biofilm's rheology is strain rate dependent, and our results suggest that this dependency is reduced due to treatment with divalent ions [63]. We propose the ionic crosslinking reduces biofilm normalized energy dissipation (Supplementary Fig. S8), making it more susceptible to strain rate yielding as indicated by our transient phase angle analysis. The rheological behaviour explored here suggests that biofilm removal strategies should be tailored to specific biofilm rheology. For example, ionic crosslinked biofilms could be susceptible to removal using high-frequency, low-amplitude deformation. Whilst, softer biofilms would be more susceptible to low frequency or sustained high amplitude deformation.

Our measurements indicated the impact of valency on stiffening, as the trivalent ion FeCl_3_ had an even greater stiffening effect when compared to FeCl_2_ (Supplementary Fig. S9, [22]). Prior studies have linked biofilm stiffening to the ionic radii of cations, with the smaller ions like Fe^2+^ (∼75 pm) inducing greater stiffening effects [64] compared to Ca^2+^ ions (∼100 pm). The chemical response of the *P. fluorescens* biofilm used in our studies agreed with these previously identified trends in *B. subtilis* and *E.coli*. However, the compositional diversity of biofilm ECM makes generalisation of prior studies and ours challenging. For example, in *B. subtilis* and *E. coli* biofilms CaCl_2_ has negligible effects upon biofilm stiffness [22,23]. While our findings focus solely on examining the exogenous addition of chemicals on the biofilm viscoelasticity, other studies have found that microbial adaptation in form of ECM secretion as well as nutrient or CaCl_2_ concentrations are important factors that determine biofilm rheology [65,66].

In the large shear regime, Fe^2+^ ions caused a characteristic two step yielding signature. Similar two-step yielding characteristics have been observed in colloidal suspensions such as silica nanoparticle dispersions and deformable microgels [[67], [68], [69]]. Two-step yielding arises due to the presence of at least two different length scales within the same material. Such interactions are often induced by increasing the ionic concentration of the dispersion medium which leads to two distinct yielding states. The first is *α* relaxation where short-range inter-particle bonds are broken, which breaks down the material into distinct clusters. Whilst the second step is *β* relaxation, where the clusters and the longer range structure are broken down [49,70]. In our FeCl_2_ treated biofilm system, the initial yield point is hypothesized to result from the breaking of cell-cell bonds within local bacterial clusters or the disruption of cell-ECM bonds. The second yield point may be due to the break-up of larger bacterial clusters, a characteristic also observed in various colloidal systems [39,50,51]. It has been found that pH can also impact the structure of the ECM network in biofilms [71,72] which can effectively change the viscoelasticity of the system. In *P. fluorescens* biofilms, the value of pH has been found to vary between 5 and 8, as we travel from the interior to the exterior of colonies [73]. Therefore, interaction between *P. fluorescens* biofilm and slightly acidic or basic solutions (Supplementary Table S1) used in our study are anticipated to cause weak swelling or deswelling effects.

Our findings align with previous observations on effects of urea on biofilms. In particular, urea has been reported to cause biofilm swelling and weakening in *S. epidermidis*, *P. aeruginosa* and multispecies biofilms [74,75]. Urea, as a chaotropic agent disrupts hydrogen bonding and protein stability within biofilms [75]. Our rheological measurements suggest that urea treatments caused softening (low G′) and swelling of the biofilm structure. Furthermore, exposure to urea significantly reduced the biofilm area fraction, which we hypothesize is due to the disruption of hydrogen bonding within the biofilm matrix. Theoretically, this could increase the biofilm equilibrium swelling point and ECM solvent concentration [76]. In the present study, the non-linear rheology of the urea biofilms mirrored the ddH_2_O biofilm. This suggests that the ECM of both biofilms were broadly similar, with the reduction in elastic and viscous moduli and increased dissipation upon yielding predominantly attributed to an increased solvent concentration. Additionally, we speculate that cononsolvency-mediated effects may be present, where urea leads to initial polymer chain collapse followed by re-swelling, an effect observed in PNIPAM polymer colloidal gels [77].

## 5 Conclusion

The results suggest that the nature of the chemical treatment can cause a predominance of either strain or rate-dependent viscoelastic effects in *P. fluorescens* biofilms. Such information can be exploited for developing biofilm removal strategies in healthcare and environmental contexts. In addition, the ability to control or induce two step yielding in biofilms using chemical treatments could open up avenues for development of bio-inks for 3D printing biofilm structures.

## CRediT authorship contribution statement

**Samuel G.V. Charlton:** Writing – original draft, Methodology, Formal analysis, Data curation, Conceptualization. **Saikat Jana:** Writing – original draft, Supervision, Methodology, Data curation, Conceptualization. **Jinju Chen:** Writing – original draft, Supervision, Funding acquisition, Data curation.

## Declaration of competing interest

The authors declare that they have no known competing financial interests or personal relationships that could have appeared to influence the work reported in this paper.

## Data Availability

Data will be made available on request.
