# Supplementary material for: Yielding behaviour of chemically treated Pseudomonas fluorescens biofilms
Source: Biofilm. 2024 Jul 3;8:100209. doi: 10.1016/j.bioflm.2024.100209 (PMC11279707; doi:10.1016/j.bioflm.2024.100209)
Supplement: Multimedia component 1 [file mmc1.pdf]

Supplementary Information: Yielding behaviour of chemically treated *Pseudomonas fluorescens* biofilms

Samuel G. V. Charlton[a,c], Saikat Jana[b,c], and Jinju Chen[c,d]

[a]Department of Civil, Environmental and Geomatic Engineering, ETH Zürich, Zürich 8903, Switzerland
[b]Ulster University, School of Engineering, 2-24 York Street, Belfast, BT15 1AP, United Kingdom
[c]Newcastle University, School of Engineering, Newcastle Upon Tyne, NE1 7RU, United Kingdom
[d]Loughborough University, Department of Materials, Loughborough, LE11 3TU, United Kingdom

July 2, 2024

Solution type	Run1	Run2	Run3	Mean ± s.d.
Distilled deionised water	7.56	7.55	7.54	7.55 ± 0.01
100 mM Urea	8.15	8.10	8.12	8.12 ± 0.03
100 mM $CaCl_2$	6.39	6.38	6.40	6.39 ± 0.01
100 mM $FeCl_2$	4.34	4.35	4.35	4.35 ± 0.00

Table S1: pH of the chemical solutions applied to the biofilms.

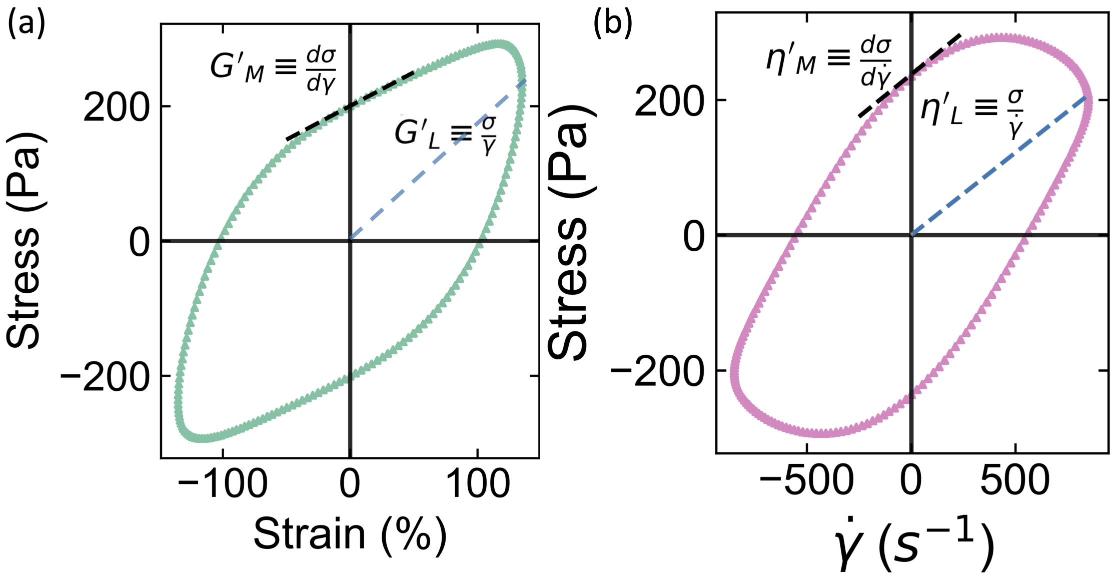

Figure S1: Figure shows (a) A plot of stress vs strain at constant frequency, also known as elastic Lissajous Bowditch (LB) plot. Minimum strain modulus (G'_M) can be calculated as tangent to the elastic LB plot at zero strain. Large strain modulus (G'_L) can be calculated as slope of the line from the origin to the point of maximum strain on a elastic LB curve. (b) A plot of stress vs strain rate at constant frequency, also known as viscous LB plot. Minimum strain viscosity (η'_M) can be calculated as tangent to the viscous LB plot at zero strain rate. Large strain modulus (η'_L) can be calculated as slope of the line from the origin to the point of maximum strain rate on a viscous LB curve.

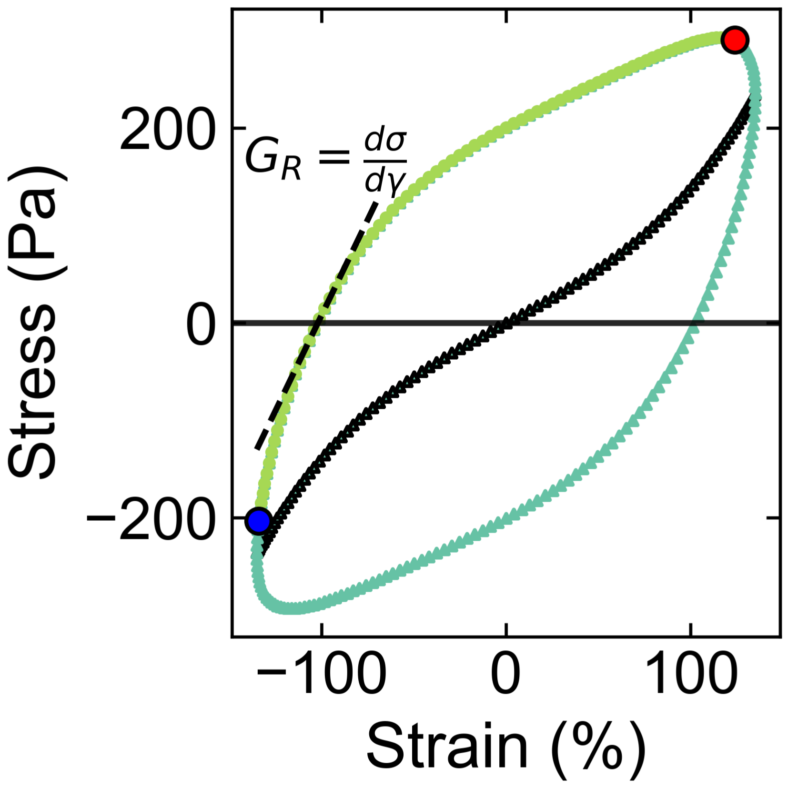

Figure S2: Figure shows an elastic Lissajous Bowditch (LB) plot that maps the intracyle stress and strain from during an oscillatory cycle. The residual modulus G_R is calculated as the differential of stress with respects to strain at the point of zero stress. Alternatively, G_R can be interpreted as tangent to the LB plot at the point where stress is zero. The accumulated strain ($\gamma_{accumulated}$) shown by light green curve, is calculated as the total stain accumulated between the lower reversal point at which strain becomes positive (the blue dot), to the point of maximum stress (the red dot).

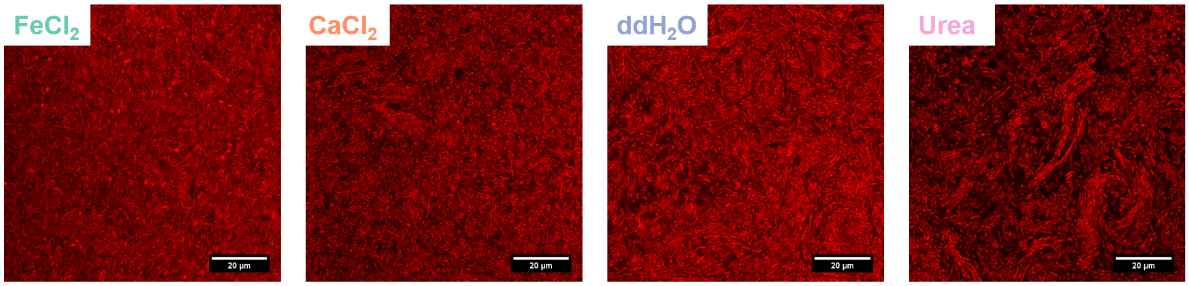

Figure S3: Confocal images of chemically treated *P. fluorescens* bacterial biofilms. Scale bar is 20 μm.

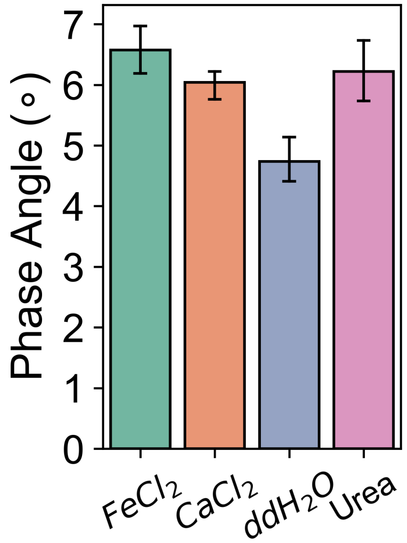

Figure S4: Phase angle ($\delta°$) of chemically treated *P. fluorescens* biofilms (determined within the linear viscoelastic region, $n \geq 3$). Error bar indicates standard deviation of mean.

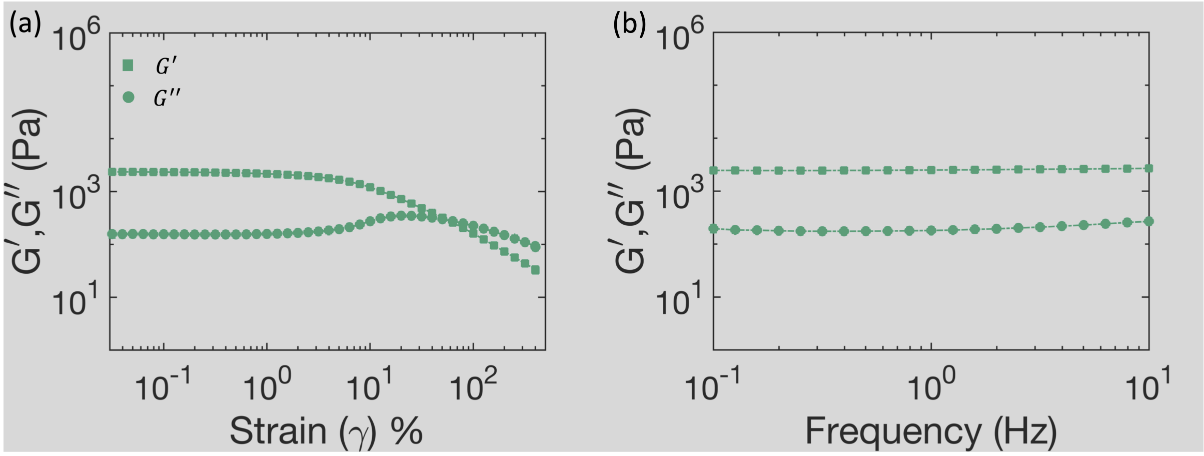

Figure S5: Viscoelasticity of biofilms grown on an agar plate with no treatments added (a) Amplitude sweep. (b) Frequency sweep , $n \geq 3$. Error bar indicates standard deviation of mean.

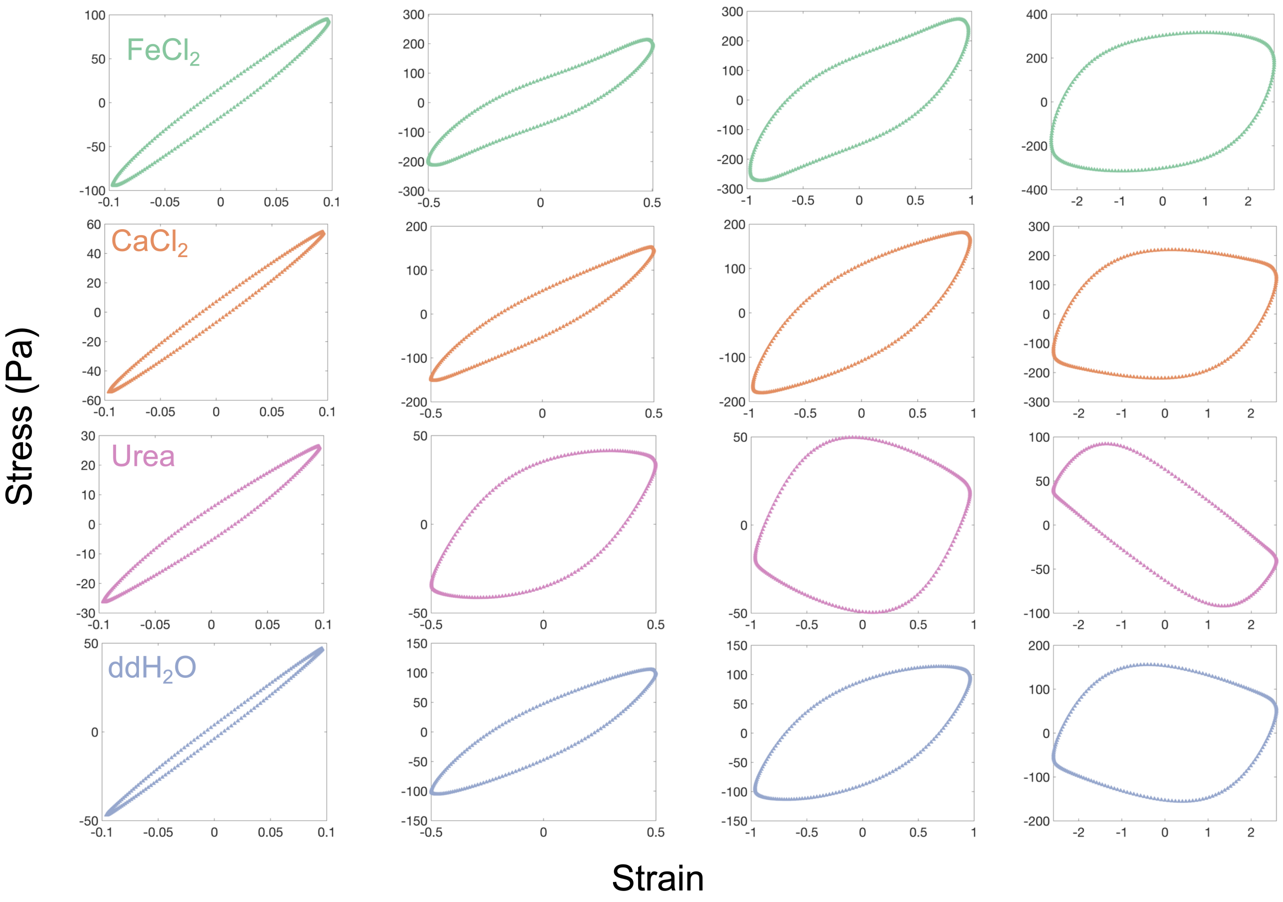

Figure S6: Elastic Lissajous-Bowditch (LB) plots for chemically treated *P. fluorescens* biofilms.

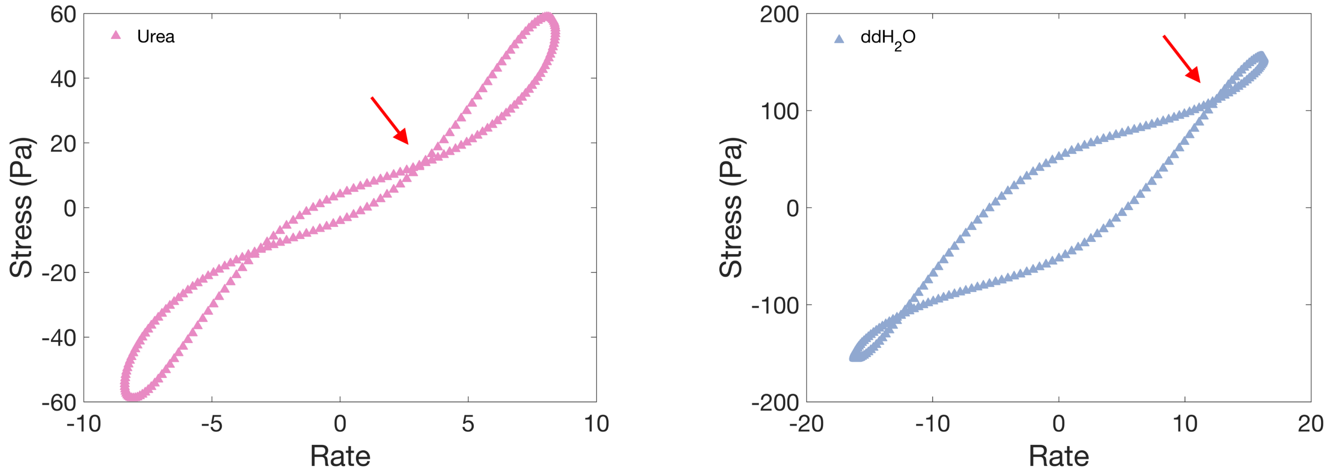

Figure S7: Viscous LB plots showing the presence of intersecting loops (red arrows) for chemically treated biofilms.

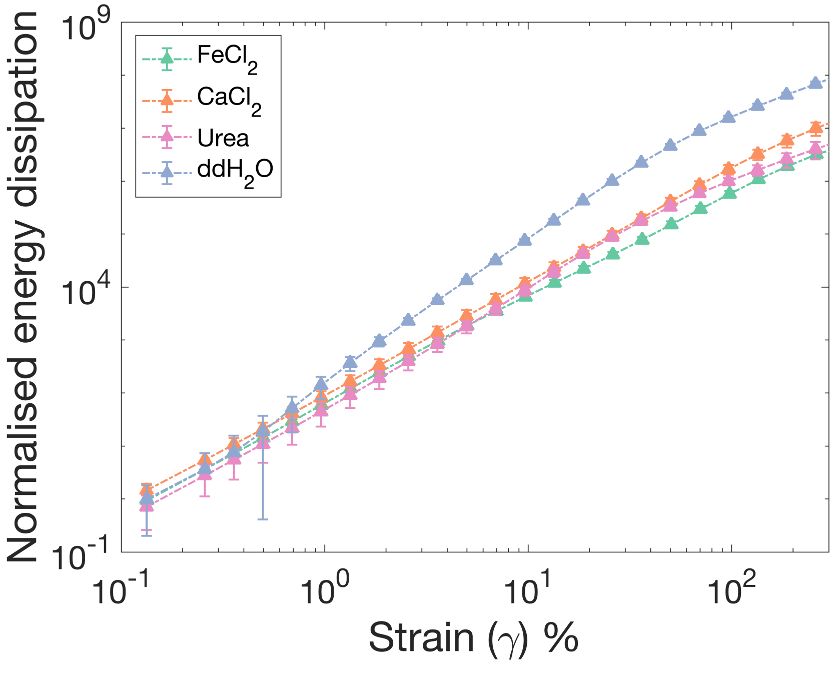

Figure S8: Normalised energy dissipated ($E_{norm} = E/E(\gamma = 0.13\%)$) as a function of applied strain. Dissipated energy (E) values were obtained by calculating area enclosed by the elastic LB plots for the applied strain (γ) and divided by the dissipated energy at $\gamma = 0.133\%$. $n \geq 3$. error bar indicates standard deviation of mean.

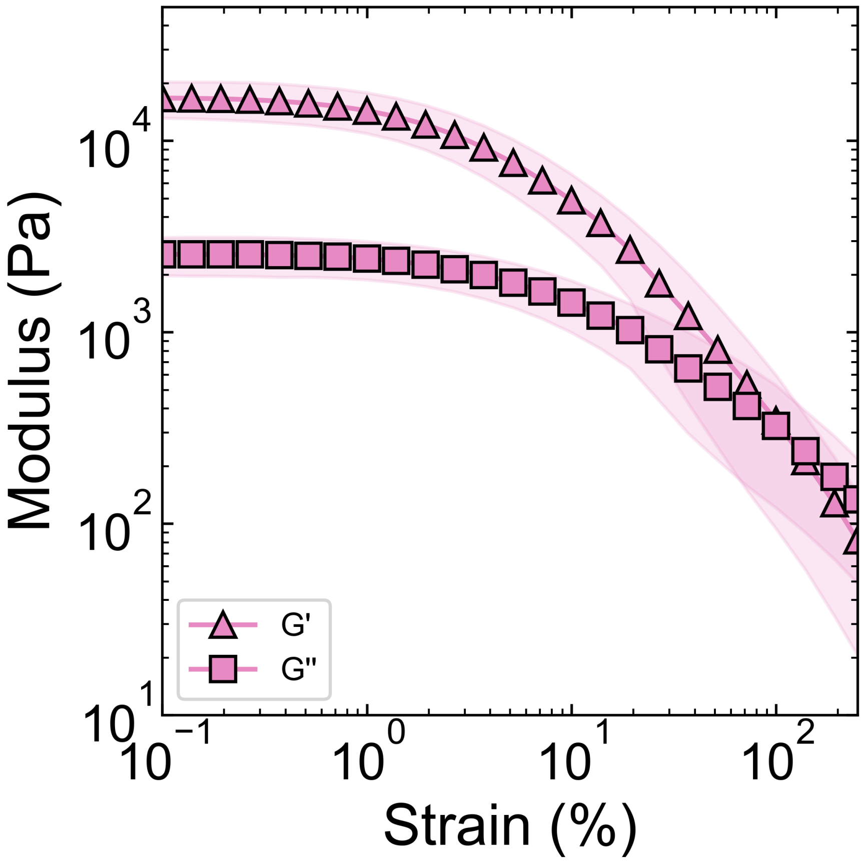

Figure S9: Amplitude sweep of *P. fluorescens* biofilms treated with Ferric chloride ($FeCl_3$), $n \geq 3$. Shaded region indicates standard deviation of mean.
